# Embodied Conversational Agents for Chronic Diseases: Scoping Review

**DOI:** 10.2196/47134

**Published:** 2024-01-09

**Authors:** Zhili Jiang, Xiting Huang, Zhiqian Wang, Yang Liu, Lihua Huang, Xiaolin Luo

**Affiliations:** 1 Department of Nursing The First Affiliated Hospital Zhejiang University School of Medicine Hangzhou China; 2 Department of Quality Evaluation Zhejiang Evaluation Center for Medical Service and Administration Hangzhou China

**Keywords:** embodied conversational agent, ECA, chronic diseases, eHealth, health care, mobile phone

## Abstract

**Background:**

Embodied conversational agents (ECAs) are computer-generated animated humanlike characters that interact with users through verbal and nonverbal behavioral cues. They are increasingly used in a range of fields, including health care.

**Objective:**

This scoping review aims to identify the current practice in the development and evaluation of ECAs for chronic diseases.

**Methods:**

We applied a methodological framework in this review. A total of 6 databases (ie, PubMed, Embase, CINAHL, ACM Digital Library, IEEE Xplore Digital Library, and Web of Science) were searched using a combination of terms related to ECAs and health in October 2023. Two independent reviewers selected the studies and extracted the data. This review followed the PRISMA-ScR (Preferred Reporting Items of Systematic Reviews and Meta-Analyses Extension for Scoping Reviews) statement.

**Results:**

The literature search found 6332 papers, of which 36 (0.57%) met the inclusion criteria. Among the 36 studies, 27 (75%) originated from the United States, and 28 (78%) were published from 2020 onward. The reported ECAs covered a wide range of chronic diseases, with a focus on cancers, atrial fibrillation, and type 2 diabetes, primarily to promote screening and self-management. Most ECAs were depicted as middle-aged women based on screenshots and communicated with users through voice and nonverbal behavior. The most frequently reported evaluation outcomes were acceptability and effectiveness.

**Conclusions:**

This scoping review provides valuable insights for technology developers and health care professionals regarding the development and implementation of ECAs. It emphasizes the importance of technological advances in the embodiment, personalized strategy, and communication modality and requires in-depth knowledge of user preferences regarding appearance, animation, and intervention content. Future studies should incorporate measures of cost, efficiency, and productivity to provide a comprehensive evaluation of the benefits of using ECAs in health care.

## Introduction

### Background

With the rapid aging of the population and lifestyle changes, chronic diseases have become a significant global public health problem, arousing great concern in people from all walks of life. In 2018, at least 1 chronic disease was experienced by 51.8% of American adults, and 27.2% dealt with multiple chronic diseases [1]. In China, chronic diseases accounted for 86.6% of total deaths and approximately 70% of the total burden of diseases [2]. Given the prolonged duration and severe health damage associated with chronic diseases, patients often require assistance in long-term care [3]. To relieve this growing burden, particularly in health care services and related costs, advancements in network communication technology have shown promise in improving the availability and quality of support services. eHealth applications allow remote patient monitoring and provide patient-tailored support in their home settings. However, many eHealth applications have faced the problem of their actual use decreasing after several weeks [4]. This decline may be attributed to the fact that most existing eHealth applications provide such support in the form of plain text or via a text-based question-answer module, whereas person-to-person interaction remains one of the best ways to communicate health information [5]. Face-to-face consultations, with the use of verbal and nonverbal behaviors such as empathy and immediacy, can foster trust and satisfaction among patients, leading to better health communication and understanding [6].

An embodied conversational agent (ECA) is a computer-based dialogue system with an embodiment that simulates a face-to-face conversation with humanlike physical properties, including verbal and nonverbal behavioral cues (eg, speech, facial expressions, and gestures) [7]. Compared with a static character image or a text-only display, the interactive, conversational modes of communication used by ECAs may potentially improve engagement by providing additional motivational and emotional support [8,9]. In health care, ECAs have been designed to assist with various tasks such as providing diabetes self-management education [10], promoting cancer screening [11], and delivering cognitive behavioral therapy for depression [12]. Despite the exciting potential for using ECAs for health purposes, their use could be ineffective or even have unintended negative consequences if the design, including visual appearance and intervention content, does not meet the user’s expectations [13]. Research has shown that design decisions related to the look and feel of ECAs significantly influence users’ psychological and emotional responses and their engagement with the applications [14]. However, how ECAs should be designed and used to maximize their effectiveness in the context of chronic diseases is still unknown. Therefore, it is crucial to systematically review the development and evaluation of ECAs in a specific context to optimize their design to provide a positive user experience and promote engagement.

Currently, there is a lack of comprehensive reviews on the development and evaluation of ECAs, particularly in the context of chronic diseases. Although there have been literature reviews on conversational agents in eHealth, they often focus on the impact of ECAs rather than the design processes involved. For example, Kramer et al [15] conducted a scoping review of ECAs in a healthy lifestyle and pointed out that the design of an ECA could have a major effect on both impact and uptake. However, reports on design activities and their results were generally incomplete or missing. Similarly, 2 other reviews by ter Stal et al [16] and Loveys et al [17] identified the most common design features of ECAs and their impact on user perception but ignored the design activities of ECAs. Another scoping review by Provoost et al [18] aimed to provide an overview of the technological and clinical possibilities of ECAs but only for patients with mental disorders. Therefore, a comprehensive literature review focusing on the design processes of ECAs in the context of chronic diseases is required.

Existing studies have primarily focused on design features rather than design processes and have examined a broader context beyond health or a specific subarea of health such as clinical psychology. In this study, we aimed to review relevant studies to understand how ECAs have been designed and evaluated specifically in the context of chronic diseases. After a preliminary exploration of the relevant literature on ECAs to determine the review method, it was found that the traditional systematic review or meta-analysis method seemed unsuitable because of the variability in populations, study designs, and measured outcomes. Compared with traditional systematic reviews, scoping reviews cover a broader range of topics in which many different study designs may be applicable, and the quality evaluation of the included research is not emphasized [19]. Therefore, we adopted the scoping review method, which provides a clear and systematic means to outline this large and diverse body of literature, using rigorous methods to minimize bias [20].

### Objectives

In this study, we undertook a scoping review focused on the development and evaluation of ECAs in the context of chronic diseases. In particular, the aims of our scoping review are as follows: (1) provide an overview of all studies on the developmental practices of ECAs for chronic diseases, (2) summarize the design and design processes of ECAs in chronic diseases, and (3) identify evaluation and outcomes reported in studies. Conducting this scoping review will benefit both technology developers and health care professionals. For technology developers, this review will provide a comprehensive understanding of the different approaches and techniques that have been used in the development of ECAs for chronic diseases, enabling them to develop a more intelligent ECA that provides a natural experience for users. For health care professionals, this review will offer actionable advice that helps them better manage and provide medical services using ECAs, ultimately improving patient outcomes.

## Methods

### Study Design

The framework for scoping reviews by Arksey and O’Malley [19] was adopted. The main five stages were as follows: (1) identifying the research questions; (2) identifying relevant studies; (3) selecting studies; (4) charting the data; and (5) collating, summarizing, and reporting the results. We followed the process outlined in the published protocol and the PRISMA-ScR (Preferred Reporting Items for Systematic Reviews and Meta-Analyses extension for Scoping Reviews) guidelines (Multimedia Appendix 1) [21].

### Identifying the Research Questions

This study mainly addressed the following questions: (1) What are the basic characteristics of the included studies? (2) How to design ECAs to guide self-care of chronic diseases? and (3) How to evaluate the impact of ECA interventions for chronic diseases?

### Identifying Relevant Studies

The databases used to locate the relevant studies were PubMed, Embase, CINAHL, Web of Science, IEEE Xplore Digital Library, and ACM Digital Library. These databases were chosen because they cover relevant aspects in the fields of health and information technology and have been used in other systematic literature reviews covering similar topics [22,23]. The search terms were identified based on a preliminary literature scan and the opinions of a research librarian to ensure a comprehensive search for relevant studies. The final search terms included an extensive list of items describing the constructs “embodied conversational agents” and “health.” A complete overview of the search terms for each construct and the inclusion criteria implemented by selecting different options and limits during the search can be found in Multimedia Appendix 2. An exemplary search strategy for PubMed is presented in Textbox 1. In addition, we used the snowball method. The search was limited to English papers published before the search date of October 1, 2023.

**The search strategy used in PubMed.**

**Health**

“Health*”[Title/Abstract] OR “mHealth”[Title/Abstract] OR “m-Health”[Title/Abstract] OR “Telehealth”[Title/Abstract] OR “Tele-health”[Title/Abstract] OR “eHealth”[Title/Abstract] OR “e-Health” [Title/Abstract] OR “Telemedicine”[Title/Abstract] OR “Tele-medicine”[Title/Abstract] OR “well-being”[Title/Abstract] OR “wellbeing”[Title/Abstract] OR “medic*”[Title/Abstract] OR “illness”[Title/Abstract] OR “patient*”[Title/Abstract] OR “disorder*”[Title/Abstract] OR “disease*”[Title/Abstract]

**Embodied conversational agents**

“Conversational agent*”[Title/Abstract] OR “Conversational assistant*”[Title/Abstract] OR “Embodied agent*”[Title/Abstract] OR “Animated character*”[Title/Abstract] OR “Animated agent*” [Title/Abstract] OR “Virtual agent*”[Title/Abstract] OR “Virtual assistant*”[Title/Abstract] OR “Virtual health assistant*”[Title/Abstract] OR “Virtual coach*”[Title/Abstract] OR “Virtual character*”[Title/Abstract] OR “Virtual human”[Title/Abstract] OR “Virtual therapist*”[Title/Abstract] OR “Virtual nurse*”[Title/ Abstract] OR “Virtual companion*”[Title/Abstract] OR “Virtual counselor*”[Title/Abstract] OR “Virtual health counselor*”[Title/Abstract] OR “Virtual clinician*”[Title/Abstract] OR “Interactive agent*”[Title/Abstract] OR “Relational agent*”[Title/Abstract]

**Combined**

1 AND 2

### Study Selection

The results of the search query were uploaded to the NoteExpress (version 3.3; Beijing Aegean Software Company) reference manager and independently assessed by 2 reviewers (ZJ and XH) to decide on their inclusion based on the title, abstract, and full text. Following an initial screening of titles and abstracts, the full texts were obtained and screened by 2 reviewers. If the eligibility of the full text was unclear, any discrepancies were reviewed by an additional author (YL) and resolved in a consensus meeting. For this review, we included chronic diseases identified by the Public Health Agency of Canada, including cancer, heart disease, hypertension, stroke, chronic respiratory diseases (eg, asthma, chronic obstructive pulmonary disease, and sleep apnea), diabetes, inflammatory bowel diseases, neurological conditions (eg, Alzheimer disease and other dementia, Parkinson disease, traumatic brain injury, and traumatic spinal cord injury), arthritis, and osteoporosis [24]. Mental illness was excluded from the list, given that support interventions of ECAs for this group may have unique features that are not generalizable to other chronic diseases. In addition, we decided to include other diseases that require self-care outside the list of the Public Health Agency of Canada, such as obesity and chronic pain. We included full papers that met the following criteria: (1) participants were adults aged ≥18 years, (2) papers were published in English, and (3) ECAs were made available to the general public (eg, general population or patient). We applied the following exclusion criteria: (1) reviews, editorials, opinions, theses, and conference abstracts; (2) papers for which full texts were unavailable; (3) ECAs used for training or educating medical professionals or not used in the context of chronic diseases; and (4) papers that did not involve ECAs (computer-generated virtual individuals with an animated appearance to enable face-to-face interaction between the user and the system) [25].

### Charting the Data and Collating and Summarizing the Results

Data extraction was conducted independently by 2 reviewers (ZJ and ZW) using an Excel (Microsoft Corp) spreadsheet. Any discrepancies in the extracted data were discussed between the authors and resolved by discussion and consensus. Extracted data included (1) paper information, (2) study information, (3) details about the ECAs (eg, identity, communication modality, and personalized content), and (4) evaluation outcomes. The content of concepts can be predefined based on the study by Kramer et al [15] (Multimedia Appendix 3). In cases where a paper included multiple studies, data extraction was performed only for the studies that met the eligibility criteria. Data were extracted separately if multiple eligible studies were included in 1 paper. Once all the study data were collected, we conducted a thematic analysis and categorized them into 3 main topics. The first topic described the identities (including ECA’s names, roles, and appearances), communication modalities, and personalization in intervention content and delivery. The second topic focused on the technologies and theories or principles used. The third topic described the evaluation measures and outcomes.

## Results

### Papers Retrieved

**Figure 1:**
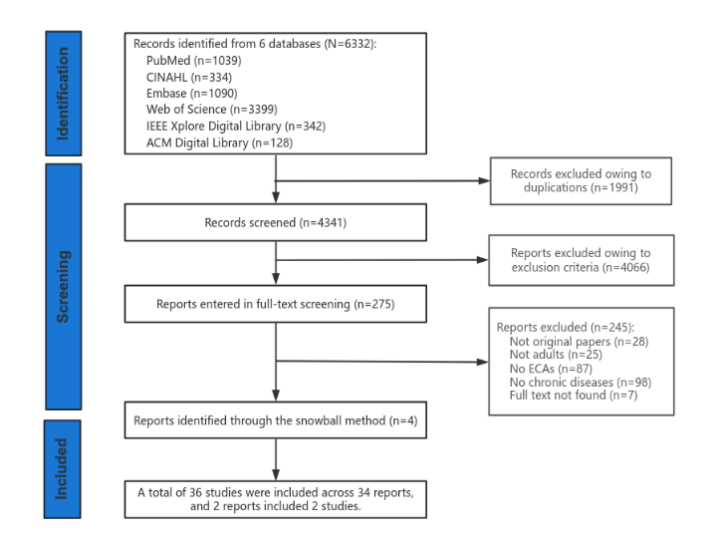
PRISMA (Preferred Reporting Items for Systematic Reviews and Meta-Analyses) flow diagram of study selection. ECA: embodied conversational agent.

The initial search identified 6332 references in October 2023; after the removal of duplicates, 4341 references (68.56%) remained. The titles and abstracts of these references were screened by both reviewers, resulting in the exclusion of 4066 references. After further evaluation, 245 papers were excluded, and the remaining 30 papers were considered eligible for a comprehensive review. In addition, 4 more studies were identified through snowballing [26-29]. This resulted in a total of 36 studies as 2 papers [30,31] included 2 studies each. Figure 1 describes the search process and outcomes.

### Description of Included Studies

Multimedia Appendix 4 [10,11,26-57] presents a summary of the studies included in this review. The characteristics of the included studies are presented in detail in Table 1.

The first study [32] was published in 2012, and 22% (8/36) of the studies were published before 2020 [27-29,32-36]. Most of the included studies were conducted in the United States (27/36, 75%) [11,27-34,36,43-57] and Australia (3/36, 8%) [10,26,37]. Other study locations included France (1/36, 3%) [38], Portugal (1/36, 3%) [39], the Netherlands (1/36, 3%) [40], and Italy (1/36, 3%) [41]. One study was conducted in 3 countries (Italy, Luxembourg, and the Netherlands) [42], and another study did not provide any information [35]. Among the studies that reported a study design, mixed method studies (8/36, 22%) [10,28,39-44] and pilot studies (8/36, 22%) [31,34,38,45-48] were the most commonly used study designs, followed by qualitative studies (6/36, 17%) [11,26,35,49-51], randomized controlled trials (5/36, 14%) [32,37,52-54], ongoing trials (4/36, 11%) [33,55-57], quasiexperimental designs (2/36, 6%) [30], longitudinal study (1/36, 3%) [29], single-group nonrandomized trial (1/36, 3%) [36], and pre- and posttest design (1/36, 3%) [27]. Of the 36 studies, 30 (83%) were conducted on patients (15/36, 42%) [10,26,32,34-37,40,42,47,52,54-57] or healthy individuals (15/36, 42%) [11,27,29-31,44-46,48-51,53] and 6 (17%) [28,33,38,39,41,43] on stakeholders such as homecare providers or experts. The mean age of the target population across studies was >40 years, except for 19% (7/36) of the studies [30,33,35,47,53,57].

**Table 1:** Characteristics of the included studies (n=36).

Study characteristics		Studies, n (%)
**Publication year**		
	Before 2020	8 (22)
	2020 or after	28 (78)
**Study country**		
	United States	27 (75)
	Australia	3 (8)
	Portugal	1 (3)
	Netherlands	1 (3)
	Italy	1 (3)
	France	1 (3)
	Italy, Luxembourg, and the Netherlands	1 (3)
	Not available	1 (3)
**Study design**		
	Mixed methods study	8 (22)
	Pilot study	8 (22)
	Qualitative study	6 (17)
	Randomized controlled trial	5 (14)
	Ongoing trial	4 (11)
	Quasiexperimental design	2 (6)
	Longitudinal study	1 (3)
	Pre- and posttest design	1 (3)
	Single-group nonrandomized design	1 (3)
**Sample population**		
	Patients	15 (42)
	Healthy adults	15 (42)
	Multiple stakeholders	6 (17)
**Targeted chronic disease**		
	Colorectal cancer	10 (28)
	Atrial fibrillation	4 (11)
	Breast cancer	4 (11)
	Type 2 diabetes	4 (11)
	Prostate cancer	3 (8)
	Chronic pain	3 (8)
	Dementia	2 (6)
	Chronic obstructive pulmonary disease and chronic heart failure	1 (3)
	Obesity	1 (3)
	Parkinson disease	1 (3)
	Sleep apnea syndrome	1 (3)
	Spinal cord injury	1 (3)
	Heart failure	1 (3)
**Main purpose of embodied conversational agent**		
	Promote screening	13 (36)
	Promote self-management	11 (31)
	Genetic counseling	3 (8)
	Therapy	3 (8)
	Assisted living	2 (6)
	Promote exercise	2 (6)
	Diagnosis	1 (3)
	Education	1 (3)
**Delivery channel**		
	Web	15 (42)
	Smartphone app	10 (28)
	Tablet	7 (19)
	Computer	4 (11)

The most common chronic condition reported in the studies was cancer, accounting for 47% (17/36) of the studies. Among these 36 studies, 10 (28%) [11,31,44-46,48-51] focused on colorectal cancer, 4 (11%) [30,43,53] focused on breast cancer, and 3 (8%) [27-29] focused on prostate cancer. Of the 36 studies, 8 (22%) addressed atrial fibrillation [34,52,55,56] and type 2 diabetes [10,26,37,39]. Other conditions included dementia (2/36, 6%) [41,42], obesity (1/36, 3%) [32], Parkinson disease (1/36, 3%) [36], sleep apnea syndrome (1/36, 3%) [38], spinal cord injury (1/36, 3%) [35], and chronic pain (3/36, 8%) [47,54,57]. One study focused on 2 diseases [40]. Of the 36 studies, 13 (36%) [11,27-29,31,44-46,48-51] used ECAs as auxiliary aids to deliver health information to promote screening. The goals of the ECA-led interventions varied, with 31% (11/36) of the studies aiming to promote self-management [10,26,33-35,37,39,40,52,55,56], including activities such as daily walking, medication taking, healthy eating, monitoring symptoms, and interaction management. ECAs were also used for mental therapy (3/36, 8%) [47,54,57], genetic counseling (3/36, 8%) [30,53], assisted living (2/36, 6%) [41,42], diagnosis (1/36, 3%) [38], and education (1/36, 3%) [43]. Of the 36 studies, 2 (6%) [32,36] used ECAs to promote exercise. ECAs were delivered through a variety of means in the included studies, with the majority being deployed on web-based platforms (15/36, 42%) [31,32,43-51,53,54,57], followed by smartphone apps (10/36, 28%) [10,11,26,33,34,37,38,52,55,56], tablet-based systems (7/36, 19%) [27,29,36,39-42], and computer-based systems (4/36, 11%) [28,30,35,46].

### Design and Design Processes of ECAs

#### ECA Identities and Communication Modalities

Across 67% (24/36) of the studies, we found that ECAs were given 9 different names, including ALEX [31,45,46,48,51], Tanya [30,34,35,52,53], Danya [43], Anne [41,42,47,57], Sylvia [40], Laura [10,26,37], Vitoria [39], iHeartHelper [33], and Ellie [54]. Of 36 studies, 14 (39%) reported that ECAs assumed the role of virtual health coaches or counselors [10,26,30,32,34-37,43,47,52,53,57], whereas others described them as virtual health assistants (n=9, 25%) [11,31,33,39,44,48-50], virtual clinicians (n=3, 8%) [45,46,51], health providers (n=2, 6%) [27,29], a virtual human interviewer (n=1, 3%) [54], and a semiexpert (n=1, 3%) [40]. Of the 36 studies, only 1 (3%) [40] provided basic information about the ECA, such as age, place of residence, and education level. In terms of embodiment, ECAs were typically presented as middle-aged women, as shown in the screenshots provided in 64% (23/36) of the studies [10,26,30,32-43,47,52-57]. The visual appearance of ECAs varied across studies. Of the 36 studies, 6 (17%) offered 4 different ECAs, whose appearances could be matched to the participants’ race and gender [11,31,45,46,48], and 3 (8%) studies [27,28,51] reported that ECAs had the appearance of an African American man. Of the 36 studies, 5 (14%) [44-46,50,51] reported that the visual appearances of ECAs were based on formative focus groups and think aloud interviews with the target population, improving the details of the character’s appearance (eg, adding white coats, removing white hair, and changing hairstyles). Of the 36 studies, 3 (8%) used a 3D model of a digital examination room as the virtual environment behind the ECA, resembling local clinical rooms [31,45,46].

Regarding the communication modalities of ECAs, 14 agents were able to communicate with users through verbal and nonverbal behaviors [10,27,30,32,33,35,36,38,39,41,45,54,​^55^,^57^]. Nonverbal behavior included facial displays of emotions, gaze shifts, eyebrow raises, head nods, body posture shifts, and hand gestures [30,37,53]. The specific presentation of these conversational modalities was detailed in 33% (12/36) of the studies. For example, 3% (1/36) of the studies reported that an agent with 2D animation would blink her eyes every 10 seconds and move her mouth for a fixed period after a new sentence appeared on the screen [40]. Another study mentioned that the virtual human had an idle breathing animation in a sitting posture and featured high-fidelity voices recorded by professional voice talents of the same race and gender [31]. To address communication shortcomings, such as lower eyesight accuracy or hearing impairment, the development of the interface took into account the needs of patients. This included customized speech speed [33] and text captions of the audio with the recommended font size [31]. To address safety concerns, user contributions to the dialogue were fully constrained and made by selecting an utterance from a menu [30,43,53]. Users had the option to respond to the agent by speaking, inputting an SMS text message, or touching an option on the screen in 8% (3/36) of the studies [10,26,37]. Only 1 ECA was designed to portray a listener responsive to the respondent’s nonverbal speaking behavior [54].

#### Personalization in Intervention Content and Delivery

Personalization was a common feature in the ECAs used in the studies to customize the content and delivery to suit individual users. This involved addressing users by their names and appropriate time contexts [34,52]. In addition, reminders, warnings, or alerts were provided based on individually reported data [55], and feedback on current progress toward the set goals was given [36]. Out of the 36 studies, in 1 (3%) study, users could respond to ECA’s queries using forced-choice text options that would trigger different responses, allowing the system to interact responsively in a personalized manner with the users [57]. The personalization in other studies was achieved based on various channels of information. Out of the 36 studies, 3 (8%) personalized the script logic based on the clinical targets provided by users’ health care professionals and users’ responses during the interactions [10,26,37]. To tailor the therapy for each patient, the ECA utterances, the patient’s responses, custom goals, and overall objective metrics such as time in the simulation were stored in an SQL database [47]. Only 2 (6%) out of the 36 studies provided dynamic adaptive genetic counseling for breast cancer based on the user model’s current state and the discourse context [30,53].

#### ECA Technology and Theories or Principles

From a technological perspective, the physical appearances of ECAs were primarily created using 3D character modeling and animation software, such as the Unity 3D (Unity Technologies) game engine [30,35,38,39,47,53], iClone (Reallusion) [28], and Fuse (Adobe Systems) [31]. Only 1 study used 2D animation implemented with scalable vector graphics and HTML animations [40]. Regarding communication modalities, speech recognition technology was used in 17% (6/36) of the studies to allow users to answer the agent’s questions orally [10,26,37,38,41,42], whereas speech synthesis technology was used to generate the agent’s spoken responses [30,34,35,39,41,42,52,53,56]. For example, a text-to-speech software, such as Speech2Go (Harpo), was used to convert written dialogues into audio files [39]. Nonverbal behaviors of the ECAs were generated using the Behavior Expression Animation Toolkit text-to-embodied speech engine [30,35,47], and the LipSync Generator was used to synchronize the agent’s lip movements with the spoken words [39]. Motion capture technology was used in 6% (2/36) of the studies to record the voice and gestures of a real person, adding a level of realism to the ECA’s behavior [28,38]. Various approaches were used in dialogue management. These included using a rules engine to determine the agent’s responses based on contextual information [39], a hierarchical transition network–based dialogue engine [35,47,53], and a scenario manager based on decision trees [38]. In addition, 8% (3/36) of the studies used voice recognition with prescripted conversational elements and a sophisticated script logic [10,26,37].

Most ECAs in the studies applied theories or therapy-derived principles to guide their content, visual design, and communication strategies. Table 2 shows 13 different theories and principles mentioned across 14 studies. Of these 14 studies, 4 (29%) incorporated behavior change theories to guide intervention content and delivery [32,33,37,39] and 10 (71%) based their interventions on theories derived from communication [30,53], technology [27,28,44,50,51], or psychological domains [27,28,32,37,49,53]. A total of 11 studies have reported the role of theory in design processes [27,28,30,37,39,44,49-51,53]. For example, the design of the software prototype intervention and dialogue creation was guided by the Behavior Change Wheel [39].

**Table 2:** Theories or principles informing the embodied conversational agent (ECA)–based interventions (n=14).

Theories or principles guiding ECA designs	Value, n (%)
Information, Motivation, and Behavioral skills model	1 (7)
Behavioral Theory	1 (7)
Social cognitive theory	1 (7)
Technology Acceptance Model	1 (7)
Transtheoretical Model	1 (7)
Behavior Change Wheel	1 (7)
Behavior change techniques	1 (7)
Unified Theory of Acceptance and Use of Technology	2 (14)
Cognitive Theory of Multimedia Learning	3 (21)
Gamification elements	2 (14)
Heuristic-Systematic Model	1 (7)
Risk Communication theories	3 (21)
Modality, Agency, Interactivity, and Navigability Model	2 (14)

### Evaluation Measures and Outcomes

#### Overview

The studies included in this review reported on both acceptability and effectiveness. Acceptability refers to the emotional attitude toward new digital health interventions, use intentions, actual use, and satisfaction [58]. Effectiveness refers to the effect of ECA-based interventions on health-related outcomes. A total of 4 studies only described protocols [33,55-57], which were not considered in this section.

#### Acceptability

A total of 31 studies reported the acceptability of ECAs. Of the 31 studies, 8 (26%) measured acceptability using validated questionnaires or adapted versions of previously published questionnaires [27,32,38,39,41,42,44,47]. The System Usability Scale was used in 13% (4/31) of the studies to measure the acceptability of the entire system [39,41,42,47]. Other instruments used included the Acceptability E-Scale [38], the Almere Model [41,42], the closeness scale [41,42], the Technology Acceptance Model [47], the Computer Self-Efficacy Scale [27], the Working Alliance Inventory [32], and the ECA Trust Questionnaire [38]. For example, 1 study assessed participants’ perceived ease of use of the system using a 24-item scale adapted from the Unified Theory of Acceptance and Use of Technology [27]. Another study used the Portuguese version of the System Usability Scale and calculated an aggregate average score of 73.75, corresponding to a borderline rating of excellent [39]. A total of 2 studies on dementia illustrated that ECA Anne received a mean score of 66.2 [42] and 67.1 [41]. In addition to questionnaires, customized items were used in 14 studies to assess users’ satisfaction with ECAs and the overall systems [10,27,29-31,34,36,38,40,43,45-48,53,54], whereas interviews and focus groups were conducted in 18 studies to explore more topics [10,11,26,28-31,35,39-44,49-52]. User satisfaction concerns items related to constructs such as liking, trust, ease of use, and desire to continue using the ECA; for example, “How much did you like Tanya?” [30]. Objective measures of user engagement with ECAs were reported in 8 studies [32,34,36,37,40-42,52]. These measures included the number of log-ins to the agent application, the time and number of interactions with the ECAs, and the time of program use. For example, 1 study showed that the time for participants to use relationship agents ranged from 3 to 30 days, and the number of log-ins to the relational agent ranged from 4 to 43 days [34]. Telemetry data were used in 2 studies to detect problems and evaluate the status quo [41,42]. In total, 2 studies measured use over time, all showing a decrease [37,40]; for example, “the program use, including the number of chats and number of blood glucose uploads, reduced over time of the program access.”

#### Effectiveness

The effectiveness of ECA intervention was evaluated in 16 studies [27,30-32,34,36,37,43,46-48,52-54]. Behavioral outcomes were examined in 4 studies [32,34,36,52]. Participants in the intervention arm showed higher daily activity and adherence to therapy than those in the control arm [52]. Gait speed and the 6-minute walk test significantly improved after the intervention (all *P*=.02) [36]. A total of 5 studies assessed participants’ knowledge using questionnaires, and it was found that their cancer knowledge significantly improved after the intervention [27,30,43,53]. Participants in the adaptive condition had significantly greater knowledge gain than participants in the nonadaptive condition and control condition [53]. A total of 3 studies assessed changes in quality of life using questionnaires [34,37,52], and 2 studies [37,52] found a positive difference in quality of life levels between participants who engaged with the ECA and those who did not. However, there was no significant difference in glycated hemoglobin change between participants in the intervention and control groups [37]. For motivational outcomes, 3 studies assessed changes in users’ motivation [27,32,34]. Self-efficacy [27,32] and patient activation [34] were assessed using questionnaires. Symptom improvements were assessed in 3 studies using questionnaires [37,47,54]. The results showed that the use of ECAs led to a greater reduction in pain interference and a marginally greater reduction in pain intensity compared with standard interviews [54]. Safety was assessed in 1 study, where participants were asked standardized questions about adverse events such as falls, diseases, injuries, and the use of any medical services [36].

## Discussion

### Principal Findings

This scoping review specifically targeted ECAs applied for chronic diseases in health care, as we aimed to inform technology developers and health care professionals of the technological possibilities and the evidence base. Our scoping review identified 32 studies and 4 ongoing clinical trials, with most papers published from 2020 onward. The most commonly reported chronic diseases were cancer, atrial fibrillation, and type 2 diabetes. The review found that ECAs were predominantly defined as female coaches or counselors interacting with users through voice and nonverbal behavior. In addition, multiple technologies and theories were applied to the design activities of ECA-delivered interventions. A combination of effectiveness and acceptability was typically assessed. Results from the studies reveal that ECAs have the potential to enhance engagement in self-care for chronic diseases, although the evidence on their effectiveness remains inconclusive.

The identified studies were not geographically diverse, with 75% (27/36) conducted in the United States and none conducted in Africa or Asia. This lack of diversity in research locations limits the generalizability of the findings, as they are embedded in Western cultures. Given the global prevalence of chronic conditions and the need for health care system–specific solutions, future research should strive to include diverse geographies to ensure the relevance of interventions in different health care systems. Among the 36 included studies, 6 (17%) explored stakeholders’ opinions. It has been shown that there were very positive relationships between homecare providers’ and patients’ perceptions of virtual agents [38], mirroring the findings by Heerink et al [59], which reaffirmed the finding that social influence plays an important role in the user acceptance of a social agent. In addition, people who were retired, highly educated, and engaged with the app were overrepresented in some studies in the interviewed sample of participants [10,26,39]. Health literacy is relevant to the development, accessibility, and successful implementation of eHealth. eHealth interventions focused on health literacy have the potential to reduce disparities in susceptible populations, where limited health literacy is more prevalent [60]. To incorporate interventions into clinical practice effectively and ensure widespread adoption, it is necessary to identify the experiences and needs of stakeholders and users who are less autonomous and less experienced with technology.

Owing to the diversity of the design activities reported in the studies, it can be challenging to draw general findings. However, some commonalities can be observed regarding the identities of the ECAs. A common design feature is giving an ECA a name, which may enhance its social presence. Through the lens of the Technology Acceptance Model, social presence, or the general sense of being with another person, is relevant to patients accepting agents because perceptions of social presence can lead to a desire for future interaction [44]. Another design feature is portraying an ECA as a coach, indicating that a relaxed and nonjudgmental role may be more successful in building a supportive relationship than an authoritative role. It is important to note that preferences may vary among different patient populations, as a recent systematic review found that racial and ethnic minority groups most often prefer a paternalistic model of health decision-making [61]. The research found that the agent’s role, such as being called a virtual physician or health care assistant, influenced the user’s expectations for the agent’s appearance [31]. Some studies reported that users tested the prototype and commented on character details that informed refinement. For example, in 1 study, an ECA was regarded as a medical authority, and changes were made to enhance its appearance, such as adding a name badge, updating clothing to include a white medical coat, and adjusting perceptions of the agent’s age [44]. Most ECAs are depicted as middle-aged women, which aligns with previous reports [15]. This may be related to the gender stereotypes associated with health guidance tasks [62]. For chronic diseases involving sensitive information, such as cancer, agents of the same race and gender are often preferred. In conclusion, developers of ECAs will have to learn to determine which identities to prioritize for their ECA but can begin analyzing this by determining their target population and their specific contexts.

Regarding communication modalities, ECAs reported in the literature mimic human conversation using interactive voice recognition, allowing users to interact with the system through voice rather than only navigating with the touch screen. When applied in the context of health communication, unconstrained speech input from conversational assistants has been found to pose patient safety risks [63]. In addition, the quality of automatic speech recognition and synthesis remains a technical problem, and there is room for improvement [41]. Nonverbal behavior has a deep impact on the process and outcome of communication, with approximately 65% of social meaning derived from nonverbal behavior [64]. In 1 study, an ECA lacked inflection in voice and exhibited a limited number of random body movements that did not align with the context of the conversation. This mismatch between a character’s speech and expected facial expressions and body movements can create an unnatural dissonance and affect acceptability, known as the “uncanny valley” [65]. Perfecting natural communication via congruence between verbal and nonverbal cues is critical [66]. This requires understanding natural behaviors and the biological processes underlying them, so as to develop efficient algorithms to implement a convincing simulation via ECAs.

Our scoping review showed that various forms of personalization are used for building content and delivery. The most common approach to personalization is providing more specific feedback based on user responses and health-related data. In this review, examples of conversational adaptation included using an individual’s current knowledge state, preferred information processing method, and other user traits such as health literacy and breast cancer risk level according to the user models [30]. From a technical point of view, future personalization may involve tailoring interventions to each user individually or even to their current status at the moment of interaction [67]. For example, Sripian et al [68] developed methods to measure and estimate the user’s emotions based on biological information. Using these methods, the ECA could react to negative user’s emotions and provide assistance. Johal et al [69] used a multisensory environment to detect when a user would like to interact with a device. Promoting the health and well-being of people through human-centered technologies requires the partnership of research networks, medical scientists, technology developers, patients, and their formal and informal caregivers.

A total of 13 theories and principles were applied to the included studies to create ECA-based interventions. However, it is difficult to determine which theory or principle is most suitable in the context of chronic diseases, which is consistent with the findings of Kramer et al [15]. Different theories and principles may need to be applied in different situations. Behavior change theory is usually combined with various behavior change technologies to guide conversational scripts and algorithms. The Modality, Agency, Interactivity, and Navigability Model, used in 2 studies, is an organizational framework for designing a multimedia learning environment. It helps to understand how interface features affect the user’s psychology through 4 affordances [50,51]. For example, navigability refers to the user’s ability to access information and complete tasks, which can aid developers in designing the navigation structure and interface layout of ECAs (eg, clear menus, navigation options, and visual cues), making it easier for users to find the desired information and functionalities. The cognitive theory of multimedia learning, which is a more recent iteration of the cognitive load theory, is also mentioned. The cognitive theory of multimedia learning recognizes that the capacity to process and store new information is limited, and presenting multimodal information redundantly may overload the cognitive capacity [70]. ECAs can be considered as a form of multimodal learning, using multiple sensory channels to enhance learning and information processing. In addition to information processing theories, future research may adopt other interpersonal communication theories to maximize the persuasiveness of ECA, as tailoring to other constructs beyond information processing modes may improve the ECA’s effectiveness in adherence motivation [53].

The evaluation measures used to assess ECAs and their effects on chronic conditions are broad and not unified. This is similar to other reviews on this topic focusing on conversational agent–delivered mental health interventions [22,71]. Several researchers have developed surveys to measure specific issues. The perceptions of the agents are most frequently reported in evaluating ECAs; for example, “How well do the words below describe Laura?” [10]. The respondents rated a range of positive and negative traits (eg, friendly, expert, reliable, annoying, and boring) using a Likert-type scale. However, there is variation in opinions and limited evidence regarding which agent characteristics are especially important. Future ECA design studies should explore both the perception of the characteristics of the designed agent and the perceived importance of these characteristics for an agent in a specific context [40]. Research suggests that incorporating humanlike characteristics in ECA design did increase user engagement [26]. However, the program use was reduced over time of the program access, and there was a dose-response relationship between the number of chats and the change in the quality of life score [37]. The dose-response relationship between the level of app use and its effectiveness suggests that more effort is required to improve the maintenance of program use over time. Regarding effectiveness-related outcomes, improvements in behavior, knowledge, symptoms, and quality of life were observed in the reviewed studies, whereas physiological data did not show significant changes. Future studies should aim to measure the efficacy of the relational agent using objective measures rather than relying on self-reporting, which is subject to multiple biases [72]. In addition to the evaluation measures mentioned earlier, the studies reviewed in this paper did not measure the cost, efficiency, or productivity improvements associated with using ECAs in health care settings. This is a significant limitation as it prevents us from determining whether ECAs are cost-effective compared with alternative approaches or if they can enhance the work of health care professionals.

### Strengths and Limitations

This scoping review has several strengths and some limitations. One of the strengths of this study is that it offers some important insights into the condition of ECAs in health care, with a focus on design activities. The study was reported according to the PRISMA-ScR guidelines, which enhanced the quality of the review. In addition, the study selection process and data extraction process were conducted by 2 reviewers independently to reduce selection bias. Agreement between the reviewers was very good for the study of both the selection process and the data extraction process.

The comprehensive literature search included 6 databases; however, the lack of standardized terminology in this field may have resulted in the omission of some related papers. In addition, limiting the search to papers published in English may result in the exclusion of ECAs developed for chronic diseases in other languages. Finally, as this is a scoping review without synthesis of the evidence, we present the outcomes as reported by the authors of primary studies; therefore, no determinations or recommendations are made regarding the appropriateness or utility of the outcomes.

### Recommendations for Future Research and Practice

One important direction for future work is to improve the interaction between ECAs and users to minimize the potential impact of uncontrolled variables (eg, user preferences) and allow for a better study of the impact of agent adaptation. First, researchers can focus on leveraging the unique characteristics of the conversational medium to personalize interactions. For example, capturing prosodic features in users’ speech can be used to automatically detect changes in mood or speech pathologies. This information will allow ECAs to provide adaptive information and services tailored to the user’s needs. Second, the behavior data of users can be technically collected and analyzed. Compared with the data collected by the questionnaires, this data-driven quantitative approach is mediated through data objectively describing their behavior, and the domain behind the data is qualitatively understood through interviews [42,73]. Finally, larger longitudinal studies need to be conducted to measure the effects of ECAs on specified subgroups over time, which will help to identify the components of the multifaceted intervention contributing to increased acceptability and positive outcomes.

### Conclusions

This scoping review followed a strict methodology to summarize and discuss the development and evaluation of ECAs in chronic diseases. The findings showed that the ECAs have been increasingly used in recent years and may have significant potential to deliver effective health interventions to promote self-care. However, there is a requirement for technological advancements in embodiment; communication methods; personalized strategies; and a deeper comprehension of user preferences regarding appearance, animation, and personalized content. In addition, there is a lack of reliable and comparable evidence in user-centered evaluation approaches. Future studies should incorporate cost, efficiency, and productivity measures to provide a comprehensive evaluation of the benefits of implementing ECAs in health care.

## Appendix

Multimedia Appendix 1 PRISMA (Preferred Reporting Items of Systematic Reviews and Meta-Analyses) checklist.

Multimedia Appendix 2 Search terms and database search.

Multimedia Appendix 3 Data extraction form.

Multimedia Appendix 4 Description of the included studies.

## Abbreviations

ECA: embodied conversational agent
PRISMA-ScR: Preferred Reporting Items of Systematic Reviews and Meta-Analyses Extension for Scoping Reviews
