# Supplementary material for: Embodied Conversational Agents for Chronic Diseases: Scoping Review
Source: J Med Internet Res. 2024 Jan 9;26:e47134. doi: 10.2196/47134 (PMC10806449; doi:10.2196/47134)
Supplement: Multimedia Appendix 2 [file jmir_v26i1e47134_app2.docx]

## Multimedia Appendix 2. Search terms and database search

**Table 1.** Search terms

| **Embodied conversational agents** | **Health** |
| --- | --- |
| Conversational agent* | Health* |
| Conversational assistant* | mHealth |
| Embodied agent* | m-Health |
| Animated character* | Telehealth |
| Animated agent* | Tele-health |
| Virtual agent* | eHealth |
| Virtual assistant* | e-Health |
| Virtual health assistant* | Telemedicine |
| Virtual coach* | Tele-medicine |
| Virtual character* | wellbeing |
| Virtual human | well-being |
| Virtual therapist* | medic* |
| Virtual nurse* | illness |
| Virtual companion* | patient* |
| Virtual counselor* | disorder* |
| Virtual health counselor* | disease* |
| Virtual clinician* |  |
| Interactive agent* |  |
| Relational agent* |  |

**Table 2.** Options and limits selected per database.

| **Database** | **Field** | **Options and limits** |
| --- | --- | --- |
| PubMed | Search in | Title/Abstract |
|  | Language | English |
| Embase | Search in | Title or Abstract |
|  | Language | English |
| CINAHL | Search in | Abstract |
|  | Language | English |
| Web of Science | Search in | Topic |
|  | Language | English |
| ACM Digital Library | Search in | Abstract |
|  | Language | English |
| IEEE Xplore Digital Library | Search in | Abstract |
|  | Language | English |
