# Supplementary material for: Embodied Conversational Agents for Chronic Diseases: Scoping Review
Source: J Med Internet Res. 2024 Jan 9;26:e47134. doi: 10.2196/47134 (PMC10806449; doi:10.2196/47134)
Supplement: Multimedia Appendix 3 [file jmir_v26i1e47134_app3.docx]

## Multimedia Appendix 3. Data extraction form

**Table 1.** Categories, components and definitions used for data charting.

| Category and Component | Definition |
| --- | --- |
| **Article Information** |  |
| Title | The title of the study |
| Authors | The first author of the study |
| Year of Publication | The year in which the study was published |
| Publication Medium | The medium in which the study was published (eg, journal article or conference paper) |
| **Study Information** |  |
| Study Country | The country in which the study took place |
| Aim | What the study aimed to find out |
| Study Design | Method used to evaluate the study |
| Study Population | The demographic background of the majority of the participants |
| Sample Size/Age | The number and average age of participants in the study |
| Target Disorder | The disorder that was targeted in the study |
| Main Purpose of ECA | What it is that the ECA does in the application. |
| Delivery Channel | Device or platform on which agent is accessed |
| Main Results | The main results of the study |
| **Design and Design Process** |  |
| ECA’s Name | The name which is given to the specific ECA |
| ECA’s Role | Who or what the ECA embodiment personifies |
| Appearance | Information about the design and design process of the appearance of the ECA (eg, gender or race) |
| Communication Modality | How the ECA interacts with the user: text, voice, non-verbal language (facial expression and body language via embodiment) |
| Personalization | Information about the development process of the personalized content and delivery of the ECA |
| ECA Technology | The technical aspects of developing the ECA |
| Theory or Principle | Theories or principles used to guide the development of ECAs |
| **Evaluation** |  |
| Outcome measure | The method used to collect the data: the outcome type measured |
| Outcome | The changes or impacts of the ECA-led intervention |
